# Multiple Ensemble Neural Network Models with Fuzzy Response Aggregation for Predicting COVID-19 Time Series: The Case of Mexico

**DOI:** 10.3390/healthcare8020181

**Published:** 2020-06-19

**Authors:** Patricia Melin, Julio Cesar Monica, Daniela Sanchez, Oscar Castillo

**Affiliations:** Tijuana Institute of Technology, 22379 Tijuana, Mexico; pmelin@tectijuana.mx (P.M.); Julio.monica@tectijuana.edu.mx (J.C.M.); danielasanchez.itt@hotmail.com (D.S.)

**Keywords:** ensembles, neural networks, fuzzy logic, COVID-19 time series

## Abstract

In this paper, a multiple ensemble neural network model with fuzzy response aggregation for the COVID-19 time series is presented. Ensemble neural networks are composed of a set of modules, which are used to produce several predictions under different conditions. The modules are simple neural networks. Fuzzy logic is then used to aggregate the responses of several predictor modules, in this way, improving the final prediction by combining the outputs of the modules in an intelligent way. Fuzzy logic handles the uncertainty in the process of making a final decision about the prediction. The complete model was tested for the case of predicting the COVID-19 time series in Mexico, at the level of the states and the whole country. The simulation results of the multiple ensemble neural network models with fuzzy response integration show very good predicted values in the validation data set. In fact, the prediction errors of the multiple ensemble neural networks are significantly lower than using traditional monolithic neural networks, in this way showing the advantages of the proposed approach.

## 1. Introduction

Recently, we noticed the rapid propagation of the COVID-19 Coronavirus around the world, appearing initially in China and then spreading to neighbor countries, like Thailand, Korea, and Japan, and after that to Europe, America, and later Africa. In particular, Europe, Italy, Spain, France, the United Kingdom, and Germany have been hit hard with the propagation of the COVID-19 virus, having to this moment many confirmed cases and deaths. After that, the virus spread to the American continent, and the United States and Canada which were also hit hard with the spread of the COVID-19 virus. Finally, the virus arrived in Mexico, where it is now becoming a large problem with almost 50,000 confirmed cases as of 18 May 2020.

In relation to COVID-19 prediction, we can mention the following work. In Chen et al. [1], the authors outline the prediction of the SARS-CoV-2 (2019-nCoV) 3C-as a protease structure. In Fan et al. [2], the authors outline an approach for the prediction of the epidemic spread of the coronavirus, driven by the spring festival transportation in China. In Goh et al. [3], the authors discuss the rigidity of the outer shell predicted by a protein intrinsic disorder model with this uncovering COVID-19 infectivity. In Grifoni et al. [4], a bioinformatics approach that can predict candidate targets for immune responses to SARS-CoV-2 was presented. In He [5], the author discusses what could still be done to control COVID-19 outbreaks in addition to the usual measures of isolation and contact tracing that most countries are imposing. In Huang et al. [6], a spatial-temporal distribution of COVID-19 in China and its prediction were described. In Ibrahim et al. [7], the authors describe the prediction of the COVID-19 spike-host cell receptor GRP78 binding site. In Ivanov [8], an approach for predicting the impact of epidemic outbreaks on global supply chains with a simulation-based analysis was presented. In Li et al. [9], the authors described the propagation analysis and prediction of the real COVID-19 time series. In Li et al. [10], the authors describe a forecasting method for the COVID-19 outbreak in China with good results. In Liu et al. [11], the authors report the understanding of unreported cases in the COVID-19 epidemic outbreak in Wuhan, China, and the importance of appropriate public health interventions. In Roda et al. [12], the authors discussed in detail why it is difficult to accurately predict the COVID-19 epidemic. In Roosa et al. [13], the authors described real-time forecasts of the COVID-19 epidemic in China from February 5, 2020 to February 24, 2020 with good results. In Ton et al. [14], the authors describe the rapid identification of potential inhibitors of SARS-CoV-2 main protease by deep model docking of 1.3 billion compounds. In Wang et al. [15], the authors describe a novel phase-adjusted estimation approach of the number of Coronavirus Disease cases in Wuhan, China. In all this previous related work, we can notice that to date, only simple monolithic neural networks or deep neural models have been used for prediction. However, in this work, we are proposing a new hybrid prediction model that combines ensemble architectures of neural networks with fuzzy logic for response integration, which has not been proposed before. We believe that our model will fill a current gap in existing research, which is the lack of use of multiple ensembles of neural networks in the prediction of complex time series, like the Coronavirus data. In addition, the basic modules are based on nonlinear autoregressive neural networks and function fitting networks. The main idea is that by combining several neural predictors with fuzzy logic, we are able to manage the uncertainty of the individual networks, and the total prediction can have lower uncertainty. This is the main contribution of the paper, the proposed model with ensembles of neural networks and a fuzzy aggregator for combining the predictions of the modules to obtain an improved prediction. The fuzzy aggregator is designed in such a way as to reduce the prediction error by using the information of the individual errors of the neural predictions. The simulation results of the proposed hybrid model are very good when compared with other approaches. In summary, the new prediction model is the main contribution of the paper. However, the resulted predictions are also very important for the Government in making the appropriate decisions to optimally manage the health care system, as in the case of Mexico, but believe that it could be used in other countries as well.

The paper is organized as follows. Section 2 describes the basic concepts about nonlinear autoregressive neural networks. Section 3 outlines the fundamental definitions of function fitting neural networks. Section 4 describes the proposed hybrid method combining ensemble architectures of neural networks with fuzzy logic for response integration. Section 5 shows the knowledge representation for the fuzzy system for response integration. Section 6 shows the simulation results, and finally, Section 7 offers the conclusion.

## 2. Nonlinear Autoregressive Neural Networks

The NAR (nonlinear autoregressive) neural network uses past values of the time series to estimate predicted future values. The NAR neural network model consists of one input layer, one or more hidden layers, and one output layer. NAR is a dynamic and recurrent network with feedback connections [16]. NAR is used in one-step-ahead or multi-step-ahead time-series forecasting. The NAR model expressed mathematically is presented in the following Equation (1):

(1) y(t)=F(y(t−1),y(t−2),…,y(t−d))

where y(t) is the value of the considered time series y at time t, and d is the time delay and F denotes the transfer function [17]. In this case, two NAR networks are used in the ensemble, one with Levenberg–Marquardt and the other with Bayesian regularization training algorithms. 

**Figure 1:**
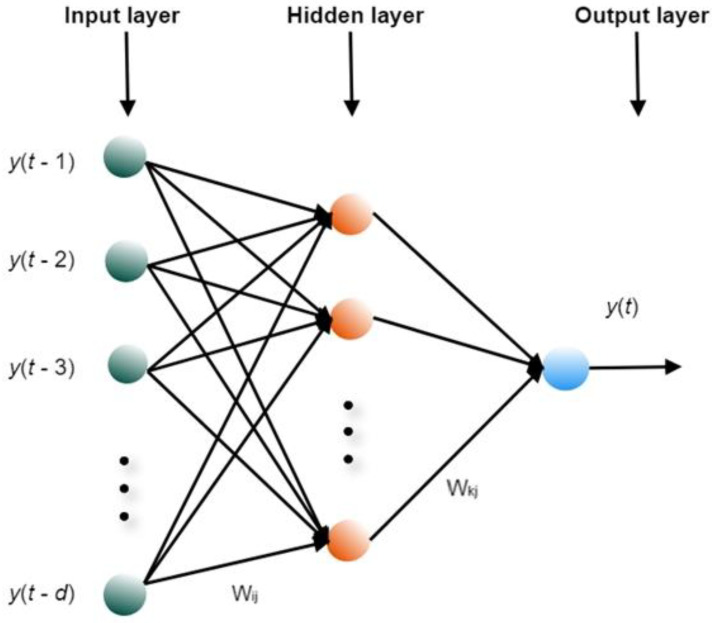
The general architecture of the NAR neural network.

In Figure 1, the NAR neural network architecture is illustrated in more detail.

## 3. Function Fitting Neural Network

**Figure 2:**
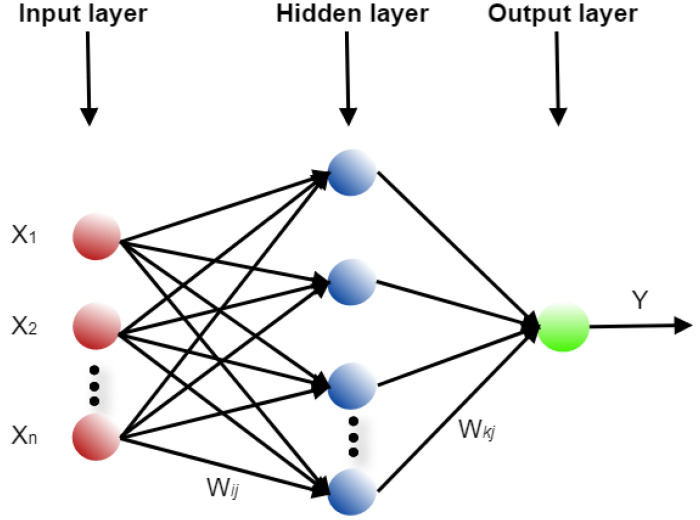
The general architecture of an artificial neural network of FITNET type.

The FITNET (function fitting neural network) is another commonly used Multi-Layer Perceptron (MLP) or a class of feedforward artificial neural network (ANN) that contains one hidden layer. A feed-forward network with one hidden layer and enough neurons in the hidden layers can fit any finite input-output mapping problem. The FITNET model uses the process of training a neural network on a set of inputs in order to produce an associate set of target outputs. The FITNET is used for curve-fitting and regression. In Figure 2, $X_{n}$ is the input neuron, $W_{ij}$ and $W_{kj}$ are the weights, n represents the neuron numbers, and Y is the neuron output [18,19,20].

In Figure 2, the general architecture of an artificial neural network (ANN) is shown.

The learning or training algorithm used in FITNET is the well-known Levenberg–Marquardt training method and the reason for this is because it is very fast for time-series data.

Neural networks, such as the NAR and the FITNET, and Fuzzy Systems, are commonly used for time-series forecasting. In fact, fuzzy systems, NAR and FITNET have been used in many areas. For example, a model was constructed for both snow-free and snowy areas to forecast monthly and daily albedo [21], wheel-wear prediction models based on NAR demonstrated being useful in predicting dynamic changes of wheel diameters [22], and FITNET was used for atomic coordinate prediction of carbon nanotubes [23]. On the other hand, neuro-fuzzy systems were used for prediction quality of a rubber curing process [24] and cardiovascular disease risk level prediction [25]. However, here the Fuzzy integrator, the NAR, and FITNET neural networks are used to help predict 10 days ahead of 12 states in Mexico and the total of the country using the confirmed and death cases of the COVID-19 using one-hidden layer. The Levenberg–Marquardt backpropagation (*trainlm*) is used as the training algorithm, the *purelin* as the transfer function and three feedback delays. The number of epochs is 500, 10 neurons in the hidden layer, and the earning rate is 0.01. The Mexican dataset was obtained from Mexico’s Government website [26].

## 4. Proposed Method

**Figure 3:**
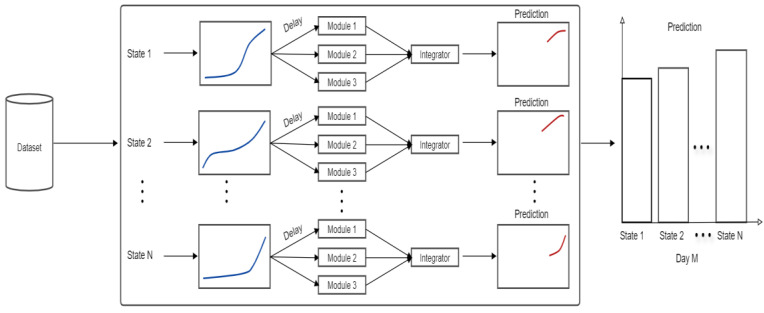
The main architecture of the system.

In Figure 3, the main architecture of the ensemble neural network model is shown. We have a dataset from COVID-19 confirmed and death cases, which consists of 12 states in Mexico and the total data of the country. In modules 1 and 2 of the ensemble, we use the NAR neural network using different parameters, and in module 3 we use the FITNET neural network to train and learn from the given information. The mean square error (MSE) of the training and actual data is normalized using Equation (2):

(2) $$MSE=\frac{1}{N}\underset{i}{\sum }\frac{{\left(P_{i}-M_{i}\right)}^{2}}{\bar{P}\bar{M}}$$

(3) $$\bar{P}=\frac{1}{N}\underset{i}{\sum }P_{i}$$

(4) $$\bar{M}=\frac{1}{N}\underset{i}{\sum }M_{i}$$

where N= the size of the training data, $x_{i}=$ the actual values, and $y_{i}=$ the trained data obtained of the sample i [27].

**Figure 4:**
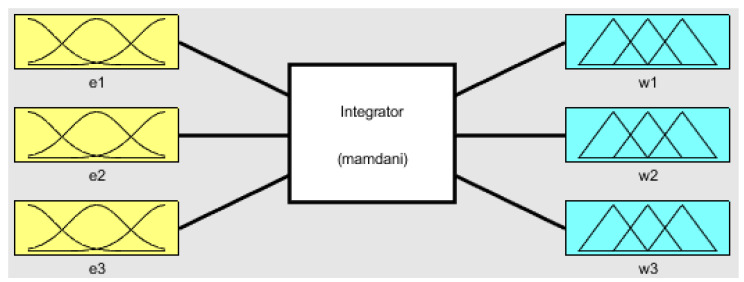
The structure of the fuzzy integrator with Gaussian membership functions.

Then the normalized mean square errors are used in the fuzzy integrator of Figure 4 to produce the weights *w*_1_, *w*_2_, *w*_3_ and then by using the expression in Equation (5) we combine the predictions to obtain the total prediction *PT*:

(5) $$PT=\frac{w_{1}p_{1}+w_{2}p_{2}+w_{3}p_{3}}{w_{1}+w_{2}+w_{3}}$$

where $w_{1}=$ weight of module 1, $w_{2}=$ the weight of module 2, $w_{3}=$ the weight of module 3, $p_{1}=$ the predicted value of module 1, $p_{2}=$ the predicted value of module 2, and $p_{3}=$ the predicted value of module 3. 

Regarding the general architecture of Figure 3, the main reasoning behind this is the following. We have one ensemble for each state in Mexico (in the Figure, this is from 1 to N). Then each ensemble has three modules, which consists of the simple neural networks (NAR and FITNET). The reason for using three modules in each ensemble is that in previous work, this architecture has provided good results. Then each ensemble has its own fuzzy aggregator to produce the final prediction of the ensemble.

The structure of the fuzzy integrator system is shown in Figure 4, which is formed by the inputs before fuzzification, the fuzzy inference system (integrator), and the fuzzy outputs after defuzzification. The inputs $e_{1}$, $e_{2}$, and $e_{3}$ consist of the normalized mean square errors of the three neural networks that have been used to predict. In this case, *e*_1_ is the MSE of module 1, *e*_2_ is the NMSE of module 2, and *e*_3_ is the NMSE of module 3. The fuzzy inference system consists of three fuzzy rules, and the three outputs are $w_{1}$, $w_{2}$, and $w_{3}$, which are obtained with the weighted mean in the defuzzification process. The main idea of this fuzzy system is to model the process of assigning the weights to the predictions of the modules according to the individual errors of the modules obtained with Equation (1). So basically, for example, if the error of module 1 is low and the errors of the other modules are high, then we assign a high weight to module 1 and low weights to the other ones. The advantage of using a fuzzy approach here with linguistic variables is that the process of assigning the weight has a level of uncertainty, which is modeled with the membership functions and fuzzy reasoning.

**Figure 5:**
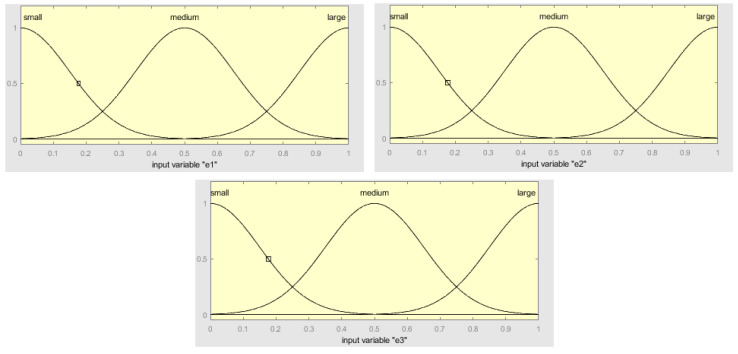
The fuzzy input membership functions of $e_{1}$, $e_{2}$, and $e_{3}$ with Gaussian functions.

Figure 5 illustrates the fuzzy inputs of the membership functions of $e_{1}$, $e_{2}$, and $e_{3}$ which is the NMSE of the neural networks in module 1, module 2, and module 3, respectively. The fuzzy values that are considered are low, medium, and large. The inputs $e_{1}$, $e_{2}$, and $e_{3}$ have been normalized in the range between 0 and 1.

**Figure 6:**
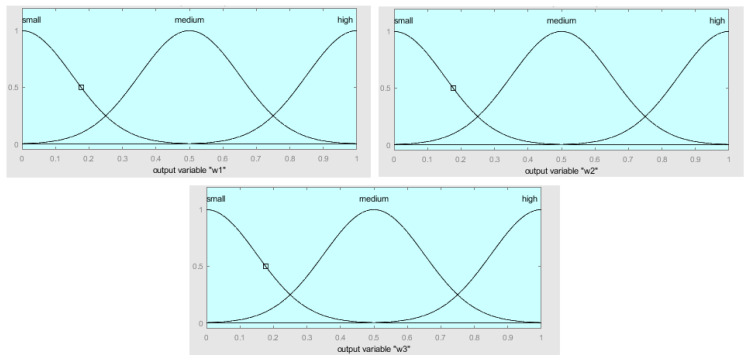
The fuzzy output membership function of $w_{1}$, $w_{2}$, and $w_{3}$ with Gaussian functions.

Figure 6 illustrates the fuzzy outputs membership functions of $w_{1}$, $w_{2}$, and $w_{3}$, which are the weighted mean errors of $e_{1}$, $e_{2}$, and $e_{3}$, respectively. The fuzzy values that are considered are low, medium, and high. The outputs $w_{1}$, $w_{2}$, and $w_{3}$ have been normalized in a range between 0 and 1.

The decision to use Gaussian membership functions was done after experimenting with Triangular, Trapezoidal, and Gaussian functions, in which better results were achieved with Gaussians. The results were better in terms of the smoothness of the output prediction results, as well as in terms of accuracy. In the paper, we only report the final design of the Gaussian membership functions that we obtained.

The fuzzy system contains three fuzzy rules, which are the following:

1. If ($e_{1}$ is small) and ($e_{2}$ is medium) and ($e_{3}$ is large), then ($w_{1}$ is high) ($w_{2}$ is medium) ($w_{3}$ is small).
2. If ($e_{1}$ is large) and ($e_{2}$ is small) and ($e_{3}$ is medium), then ($w_{1}$ is small) ($w_{2}$ is high) ($w_{3}$ is medium).
3. If ($e_{1}$ is medium) and ($e_{2}$ is large) and ($e_{3}$ is small), then ($w_{1}$ is medium) ($w_{2}$ is small) ($w_{3}$ is high).

These fuzzy rules express the knowledge of how to combine predictions based on their corresponding errors. Basically, the rules are assigning the weights (outputs) used in performing the average based on the fuzzy values of the errors in the modules. The reason for preferring Mamdani over Sugeno modeling is because a Mamdani fuzzy model is more interpretable in terms of the fuzzy rules (completely linguistic), and also is easier to design. The advantage of using fuzzy logic here is that we are able to handle the uncertainty in making a combined prediction, which is similar to combining the opinions of three experts. 

## 5. Knowledge Representation of the Fuzzy System

In this Section, we show the knowledge representation of the fuzzy system with Gaussian membership functions. The membership values for the Gaussian membership function are defined in the following Equation (6): The membership value μ(x) is the degree to which a given input x belongs to that membership function 0≤μ(x)≤1:

(6) $$\mu \left(x\right)=e^{-\frac{{\left(c-x\right)}^{2}}{2\sigma^{2}}}$$

where c= the center, and the variance σ are the design parameters.

The Equations (7)–(9) show the membership functions used for the inputs $e_{1}$, $e_{2}$, and $e_{3}$.

(7) $$Low\left(;0.15,0\right)=e^{-\frac{{\left(x-0\right)}^{2}}{2*0.15^{2}}}$$

(8) $$Medium\left(x;0.15,0.5\right)=e^{-\frac{{\left(x-0.5\right)}^{2}}{2*0.15^{2}}}$$

(9) $$Large\left(x;0.15,1\right)=e^{-\frac{{\left(x-1\right)}^{2}}{2*0.15^{2}}}$$

The Equations (10)–(12) show the membership functions used for the outputs $w_{1}$, $w_{2}$, and $w_{3}$.

(10) $$Low\left(x;0.15,0\right)=e^{-\frac{{\left(x-0\right)}^{2}}{2*0.15^{2}}}$$

(11) $$Medium\left(x;0.15,0.5\right)=e^{-\frac{{\left(x-0.5\right)}^{2}}{2*0.15^{2}}}$$

(12) $$High\left(x;0.15,1\right)=e^{-\frac{{\left(x-1\right)}^{2}}{2*0.15^{2}}}$$

The particular parameter values for the membership functions were defined considering the three possible fuzzy values, which are Low, Medium, and High, assigned and adjusted in a manual way [28,29,30,31,32].

## 6. Simulation Results

**Figure 7:**
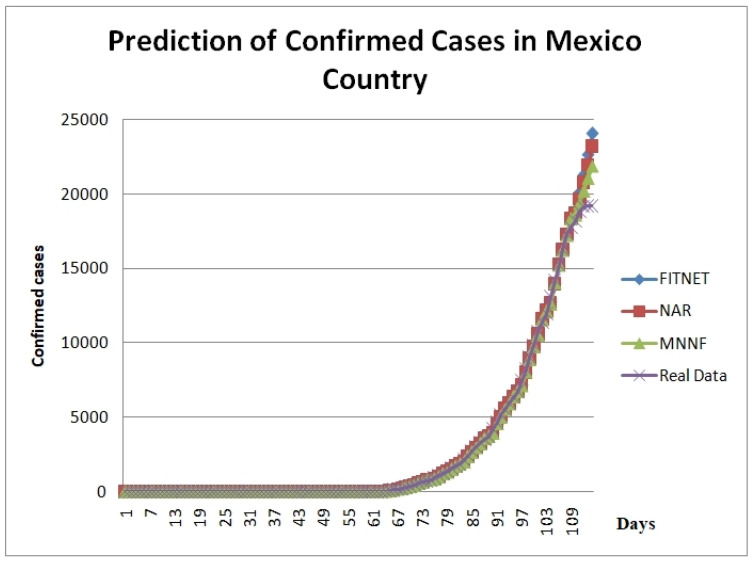
Comparison of the results of confirmed cases predicted in Mexico using different neural network models.

Figure 7 shows the comparison of results of Confirmed Cases Prediction in Mexico using different neural network models; two of them are a monolithic model, FITNET, and NAR versus the Modular Neural Network, which uses a fuzzy logic integrator. Table 1 shows a comparison of the predicted values for confirmed cases of COVID-19 in 10 days ahead for Mexico (whole Country).

**Figure 8:**
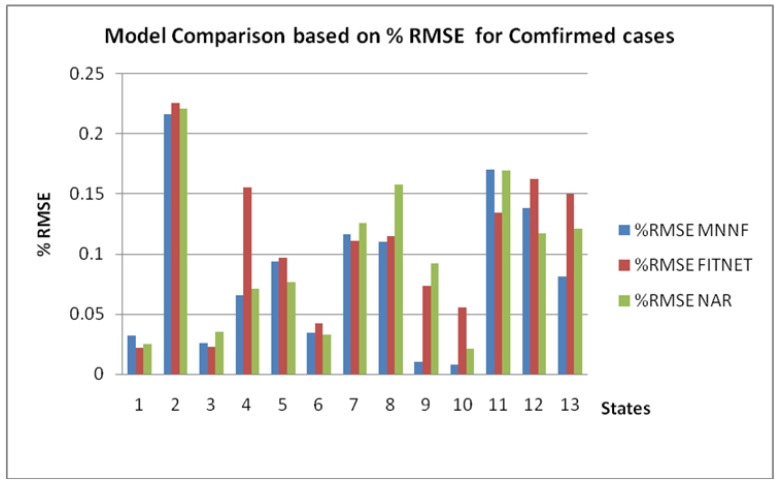
Comparison of % RMSE in confirmed cases for the different models of ANN for the 12 states.

Figure 8 shows the comparison of the % Root Mean Squared Error (RMSE) in confirmed cases for the different models of ANN for the 12 states and the country of Mexico where the states are indicated as follows: 1 is Baja California, 2 Ciudad de Mexico, 3 Coahuila, 4 Estado de Mexico 5 Jalisco, 6 Nuevo Leon, 7 Puebla, 8 Quintana Roo, 9 Sinaloa, 10 Tabasco, 11 Veracruz, 12 Yucatan and 13 the Country of Mexico. In Figure 8, we can note that the proposed Modular Neural Network with Fuzzy MNNF has lower errors. Table 2 shows the relative errors of prediction for the states and the whole country.

**Figure 9:**
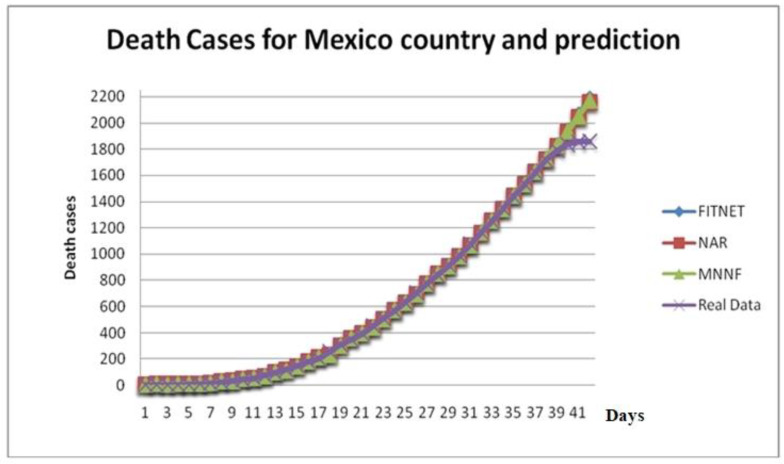
Death cases in Mexico and prediction.

Figure 9 shows the comparison of results of Death Cases Prediction in Mexico using different neural network models, two of them a monolithic model, FITNET, and NAR versus the Modular Neural Network, which uses a fuzzy logic integrator. Table 3 shows a comparison of the predicted values for death cases of Covid-19 in 10 days ahead for Mexico (the whole country). Table 4 shows relative errors of prediction of death cases for the states and the whole country.

**Figure 10:**
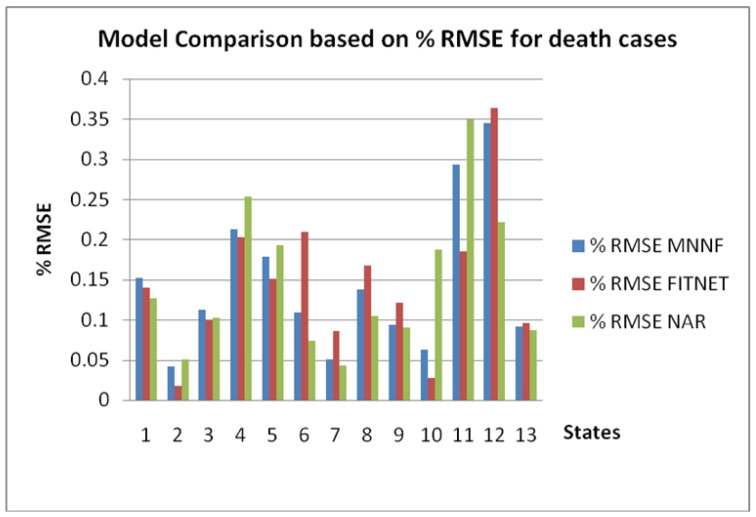
Comparison of % RMSE in death cases for the different models of NN for the 12 states and the Country of Mexico.

Figure 10 shows the comparison of % RMSE in death cases for the different models of NN for the 12 states and the Country of Mexico where: 1 is Baja California, 2 Ciudad de Mexico, 3 Coahuila, 4 Estado de Mexico 5 Jalisco, 6 Nuevo Leon, 7 Puebla, 8 Quintana Roo, 9 Sinaloa, 10 Tabasco, 11 Veracruz, 12 Yucatan, and 13 the Country of Mexico.

**Figure 11:**
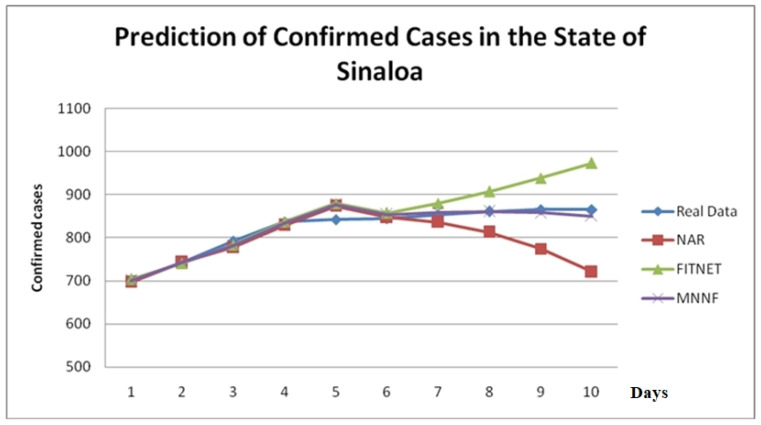
Comparison of prediction of confirmed cases for the state of Sinaloa.

Finally, we show in Figure 11 an example of the prediction in one particular state of Mexico (Sinaloa). We are predicting 10 days ahead (data not previously seen by the model), and in Figure 11 we show the results of the proposed MNNF model when compared to the NAR and FIT models. We can clearly appreciate how the proposed MNNF model is following very closely the real data and the other models after day 5, where they drift apart and loose prediction value. Our explanation of this behavior is that the proposed MNNF is using fuzzy logic for aggregating the results of the modules, and in some way, the uncertainty in making a prediction is being managed appropriately. 

## 7. Conclusions

In this paper, a new approach with multiple ensemble neural network models and fuzzy response aggregation for the COVID-19 time series was proposed. Ensemble neural networks were used to produce several predictions under different conditions. Fuzzy logic was then used to aggregate the responses of several predictor modules, in this way, improving the final prediction by combining, in a proper way, the outputs of the modules. Fuzzy logic helps in handling the uncertainty in the process of making a final decision about the prediction. The complete model was tested for the case of predicting the COVID-19 time series in Mexico, at the level of the states and the whole country. Simulation results of the multiple ensemble neural network models with fuzzy response integration show very good predicted values in the validation data set. In fact, the prediction errors of the multiple ensemble neural networks were significantly lower than using monolithic neural networks, in this way clearly showing the advantages of the proposed approach. We have to say that the proposed model can be viewed as a general prediction model because it can be applied in other time periods of the COVID-19 time series. For example, in the case of Mexico, right now the time series show an increasing trend, which is presented in this paper, but eventually, there is be a turning point, and the series will decrease, but the model will not have any problem. This is because once we have new data with a decreasing trend, we will train the simple neural networks again and use the same architecture of multiple ensembles and fuzzy aggregators to produce the new predictions in a decreasing fashion.

As future work, we plan to apply the same type of model to other COVID-19 data sets from other countries. In addition, we can also consider other time-series prediction problems, like in finance or economics. Also, regarding the model, we can optimize the structure of the neural networks using meta-heuristics, and we can use type-2 fuzzy logic in the response integration, expecting that results should improve, like in related works [33,34]. Finally, we envision improving the work in this paper by using adaptive fuzzy and neural network techniques, like in [35,36], or applying the proposed models in other kinds of applications [37,38]. 

## Figures and Tables

**Table 1:** Comparison of predicted values for confirmed cases of Covid-19.

Predicted Day	Real Data	FITNET	NAR	MNNF
1	14,230	13,988	13,988	14,035
2	15,246	15,148	15,291	15,226
3	16,252	16,216	16,298	16,226
4	17,301	17,279	17,301	17,241
5	17,783	18,391	18,386	18,333
6	18,205	18,862	18,745	18,597
7	18,850	20,045	19,678	19,391
8	19,172	21,302	20,783	20,221
9	19,220	22,637	21,953	21,053
10	19,224	24,053	23,228	21,900

**Table 2:** Example of % RMSE for the different models for the confirmed cases.

	Baja California	Cd. de Mexico	Estado de Mex	Jalisco	Nuevo Leon	Quintana Roo	Sinaloa	Mexico Country
**MNNF**								
MSE	2529.072	1,263,297.767	41,570.1111	1055.7705	131.8613	7513.0870	74.2234	2,415,010.109
RMSE	50.2898	1123.9651	203.8874	32.4926	11.4830	86.6780	8.6153	1554.0302
%RMSE MNNF	0.0322	0.2157	0.0651	0.0936	0.0343	0.1099	0.0099	0.0808
**FITNET**								
MSE:	1158.3682	1,373,761.46	235,501.924	1122.0012	198.0156	8178.0354	3994.9508	8,280,063.46
RMSE:	34.0348	1172.0757	485.2854	33.4962	14.0718	90.4324	63.2056	2877.5099
%RMSE FITNET	0.0218	0.22500	0.1550	0.0965	0.0421	0.1147	0.07307008	0.14968321
**NAR**								
MSE	1463.8333	1,318,844.292	49,312.6242	706.70452	117.464185	15,407.8063	6370.45123	5,416,634.47
RMSE	38.2600	1148.4094	222.0644	26.5839147	10.8380895	124.128185	79.8151065	2327.36642
%RMSE NAR	0.0245	0.22046	0.0709	0.0766	0.0324	0.1575	0.0922	0.1210

**Table 3:** Predicted death cases in 10 days for Mexico.

Predicted Day	Real Data	FITNET	NAR	MNNF
1	1251	1256.14422	1255.71983	1256.63828
2	1347	1339.80171	1339.88456	1340.6627
3	1438	1445.1368	1442.91771	1444.02958
4	1531	1533.02409	1532.79844	1533.68764
5	1625	1628.71662	1627.20689	1628.18016
6	1717	1724.59421	1722.99607	1723.98249
7	1788	1829.92399	1824.7057	1827.52789
8	1837	1941.17027	1930.41912	1935.97883
9	1856	2058.54869	2040.3259	2049.49095
10	1859	2182.306	2154.63482	2168.29111

**Table 4:** Example of % RMSE for the different models for death cases.

	Baja California	Ciudad de Mex	Estado de Mexico	Jalisco	Nuevo Leon	Quintana Roo	Sinaloa	Mexico Country
MNNF								
MSE:	1119.3858	202.0965	2578.2210	24.9536	2.6856	254.1817	168.0517	28,901.5512
RMSE:	33.4572	14.21606	50.7761	4.9953	1.63879	15.9430	12.9634	170.0045
% RMSE	0.1520	0.0421	0.2124	0.1784	0.1092	0.1374	0.0932	0.0914
FITNET								
MSE:	948.1897	35.1181	2342.7949	17.8936	9.8660	377.9779	283.5697	31,643.8956
RMSE:	30.7926	5.9260	48.4024	4.2300	3.1410	19.4416	16.8395	177.8873
%RMSE	0.1399	0.0175	0.2025	0.1510	0.2094	0.1676	0.1211	0.0956
NAR								
MSE:	780.6350	294.4490	3664.4998	29.1810	1.2292	146.1051	159.1990	26,297.2756
RMSE:	27.9398	17.1595	60.5351	5.4019	1.1087	12.0873	12.61744	162.1643
%RMSE	0.1269	0.0509	0.2532	0.1929	0.0739	0.1042	0.0907	0.0872
